# Pickerill’s Work on the Prevention of Caries

**Published:** 1914-03-15

**Authors:** Guy S. Millbury


### PICKERILL'S WORK ON THE PREVENTION OF CARIES.

BY DR. GUY S. MILLBURY.

Extracted from a paper read before the San Francisco Dental Society and published in the January *Pacific Dental Gazette*.

In the beginning, Pickerill touches briefly on the history of dentistry from the earliest Egyptian period, 3000 years before the Christian era, down to the present day. He discusses the subject of dental caries from racial, sexual, age, and susceptibility standpoints. The pathology of dental caries from the theory advocated by Hippocrates, viz., "that the diseases are due to a disturbance of the four principal humors of the body," down to Miller's, now commonly accepted chemicoparasitic theory, is discussed concisely.

He takes issue with J. Leon Williams and G. V. Black, on the question that the presence of bacterial plaques is an essential stage in the progress of dental caries.

In dealing with the subject of oral secretions, he maintains that it is "at the enamel surface alone, that the process of dental caries is able to be prevented, either by natural or artificial means. This point being passed, it is only a matter of time until the tooth is completely destroyed, except in the instance of arrested caries."

In his researches on enamel he has arbitrarily classified the teeth into three classes:

1st—Native—uncivilized tribes, only slightly affected with caries.

2nd—Sclerotic, (hard), characterized by hardness, yellow color, and immunity to caries.

3rd—Malacotic (Gr. Malakos, soft), characterized by whiteness, softness, and susceptibility to caries.

He states that Tomes, Black, Leon Williams, and Kirk are practically agreed in finding no appreciable difference in the hardness and softness of enamel, and he questions the customary method of determining this fact. The usual method of determining hardness is by applying a decided force and it can readily be seen that when such force is applied to iron the result would he hardly appreciable, but if the same force were applied to the diamond, the hardest known substance, it would crush or fracture into thousands of pieces. Pickerill, therefore, resorts to the geological method, that is, by scratching a given surface with a substance that is harder in character. There are usually ten classes of minerals with regard to their degree of hardness and each class will scratch the substance of the class next below it. He has also devised an instrument styled a Sclerometer, by means of which a sharp pointed steel instrument is automatically raised and lowered with a given force, determined by weights, striking the surface of the enamel at regular intervals. The depth of penetration indicates the degree of hardness of the enamels.

In discussing the resistibility of the enamel, he brings to light a new conception of its surface and presents an idea regarding the formation of the enamel, which is new. He has found that upou staining the surface with ordinary graphite and viewing it with reflected light that there are furrows on the surface of the enamel; that these furrows are deeper and wider in the malacotic than in the sclerotic; he designates these furrows as imbrication lines. Noyes in his work on the formation of the enamel does not mention these lines.

Pickerill found that in native teeth the enamel was much thicker at the gingival third or contact point than in Anglo-Saxon teeth. This provides a small interseptal space and hence less liability for the food to lodge therein.

A series of experiments with silver nitrate showed that the average penetration of the enamel by this salt was 0.27 mn. in the malacotic teeth, 0.13 mn. in the sclerotic teeth, and scarcely observable in the Maori teeth. His hypothesis, as to the function of Nasmyth's membrane, in one that will require considerable thought and elaboration before it will be generally accepted. This hypothesis interpreted is, that the membrane remains on the surface of the enamel for an indefinite period after the tooth has erupted and thereby serves as a dialyzing membrane.

Those of us who are familiar with chemistry, know that there are a number of soluble salts in the saliva, and that frequently acids are produced by the fermentation of sugars or carbohydrates taken into the mouth. The organic substances in the saliva, chiefly mucin and occasionally some albumen, are colloids. They do not readily pass through a dialyzing membrane. Crystalloids or solutions of soluble salts pass through such a membrane more readily, and acids even more readily than Crystalloids. Pickerill states that, if the acids be present in the mouth, they will pass through this membrance faster than the alkaline salts and thus have a tendency to disintegrate the tooth substance, and produce that opacity which precedes decay. If no acid be present the salts will pass through quite readily and tend to harden the surface of the teeth, and if mucin is present in large quantities, as in ropy or viscid saliva, the deposition of lime salts is hindered thereby, and the teeth are softer and more susceptible to decay.

His conclusion on the structure of the teeth that "the difference in surface structure is wholly developmental and, therefore, a more highly resistant enamel, is to be attained by a more physiological use of the jaws in early life," is indeed a logical one.

His chapter on the saliva is the most interesting part of his work. He states that opinions as to the influence of the saliva on the teeth vary in the extreme, and he claims that a fluid which constantly bathes the teeth must normally have a beneficial effect upon them. There is a general pathological law that stagnation favors decay and disease. We recognize this law in our daily life. We also find it manifested in the mouth when use, or rather disuse, of the teeth brings about a pathological condition. The flow of the saliva, constantly circulating about the mouth, overcomes this.

When we realize that from 600 cc. to 1500 cc. of saliva is secreted daily, we cannot but appreciate the value of these statements.

A most interesting thought, and one that upsets all our former ideas, is his statment that the normal secretion of the saliva is a reflex one, and can be stimulated or depressed at will. Such being the case, there is no reason in the world why we should not be in a position to help our patients in maintaining good health by educating them as to the importance of the teeth and the oral secretions, by studying the problem of diet, thus inhibiting the encroachment of diseases, which gain access to the body by the way of the oral cavity. He maintains that the food upon coming into contact with the taste buds in the tongue, send afferent impulses to the medulla via the lingual, glosso-pharyngeal, and sensory branches of the fifth nerve; the afferent impulses are returned via the chorda-tympani to the submaxillary and sublingual glands, and via the auriculo-temporal to the parotid glands. When the food comes in contact with the taste buds, thus stimulating salivary glandular activity, the secretion which follows is influenced wholly by the character of the food. For fruit acids (and we have heard much about the evil effects of eating oranges) will stimulate a markedly increased flow of alkaline saliva that is prolonged for quite a period of time after the ingestion of the fruit.. On the contrary, certain substances, such as bread and butter, have a depressing or inhibiting influence and both the quantity and alkalinity of the saliva falls below normal.

He discusses clearly the psychical influence on these glands, via the optic, olfactory, and auditory nerves. His experiments which have been verified by repetition are quite conclusive. To obtain the saliva from the parotid glands a thin walled German silver canula was inserted in Stenson's duct. During the mastication of food, the saliva from these glands was collected. Similarly a vulcanite segregatory denture was devised by him to obtain the secretion from the sublingual and submaxillary glands. He found that the parotid saliva varies in quantity from less than 1-3 to 1-30 the quantity of the sublingual and submaxillary glands. In order to give some idea as to the character of his experiments the following figures are quoted in his table of foods:

*TABLE I.

Saliva during stimulation.

| Substances masticated or used as Stimulant. | Parotid | | Submaxillary & Sublingual | | Total saliva from all Glands | | | Resting Saliva 15 min. after stimulation | | |
|---|---|---|---|---|---|---|---|---|---|---|
| | C. C. per min. | Alkalinity per C. C. | C. C. per min. | Alkalinity per C. C. | C. C. per min. | Alkalinity per C. C. | Total Alkalinity per min. Alkalinity index | C. C. per min. | Alkalinity per C. C. | Alkalinity per C. C. |
| Normal saliva resting to show increase or decrease | | | | | 1.65 | 1.05 | 1.73 | 1.65 | 1.05 | 1.73 |
| (Soft) Bread and Butter | 0.15 | 1.0 | 0.65 | 0.76 | 1.60 | 0.80 | 1.28 | 1.50 | 1.00 | 1.50 |
| Orange | 0.10 | 1.0 | 5.00 | 1.40 | 11.00 | 1.36 | 15.00 | 1.90 | 1.70 | 3.23 |
| Biscuit (soft) | 0.20 | 1.3 | 1.20 | 0.91 | 2.80 | 1.03 | 2.90 | 1.70 | 1.00 | 1.70 |
| Apple | 0.82 | 1.6 | 4.20 | 1.40 | 10.04 | 1.40 | 14.38 | 2.30 | 1.50 | 3.50 |
| Bread (dry) | 0.30 | 1.0 | 1.35 | 0.83 | 3.30. | 0.86 | 2.84 | 1.70 | 1.00 | 1.70 |
| Pineapple | 0.50 | 1.2 | 3.20 | 1.00 | 7.40 | 1.02 | 7.60 | 2.30 | 1.20 | 2.70 |
| Cake | 0.40 | 0.5 | 1.20 | 1.00 | 3.20 | 0.87 | 2.80 | 1.80 | 1.00 | 1.80 |
| Chocolate | 0.80 | 0.6 | 3.30 | 0.90 | 8.20 | 0.84 | 6.90 | 1.80 | 1.20 | 1.90 |
| "Brown" Bread | 0.10 | 1.0 | 0.80 | 1.00 | 1.80 | 1.00 | 1.80 | 2.00 | 0.70 | 1.40 |
| Banana | 0.55 | 0.8 | 1.80 | 0.75 | 4.70 | 0.76 | 3.58 | 1.75 | 1.10 | 1.90 |
| Grapes (very ripe) | 0.10 | 1.0 | 2.30 | 0.74 | 4.80 | 0.75 | 3.60 | 2.70 | 0.80 | 2.36 |
| Celery | 0.45 | 2.0 | 2.00 | 1.20 | 4.90 | 1.34 | 6.60 | 2.20 | 1.40 | 3.08 |
| Carrot (boiled) | 0.10 | 1.5 | 2.00 | 0.80 | 4.20 | 0.83 | 3.50 | 1.60 | 1.30 | 2.08 |
| Carrot (raw) | 0.50 | 1.6 | 2.50 | 1.20 | 6.00 | 1.20 | 7.60 | 1.80 | 1.70 | 3.06 |
| Figs | 0.85 | 1.0 | 2.20 | 0.90 | 6.10 | 0.92 | 5.60 | 2.10 | 1.20 | 2.50 |
| Dates | 0.30 | 1.1 | 1.80 | 0.70 | 4.20 | 0.75 | 3.18 | 2.10 | 1.10 | 2.31 |
| Meat (mutton) | 0.10 | 1.0 | 1.20 | 0.83 | 2.60 | 0.84 | 2.19 | 1.70 | 0.90 | 1.53 |
| Radish | 0.25 | 1.0 | 2.00 | 1.57 | 4.50 | 1.50 | 6.78 | ---- | ---- | ---- |
| Stewed Apple | 1.00 | 1.1 | 5.50 | 1.02 | 13.00 | 1.04 | 13.64 | 2.20 | 1.20 | 2.64 |
| Lemon | 0.10 | 2.0 | 3.30 | 1.03 | 6.80 | 1.57 | 7.19 | 5.20 | 1.20 | †6.24 |

*From Pickerill's Work, p. 137 †Two minutes afterwards.

Aside from these investigations, he found that tea had an inhibiting influence and that most of the liquors, port wine and whiskey in particular, had a stimulating influence, indicatings, to those of us who are so inclined, that a cocktail is a real appetizer.

His investigations on tooth-pastes and powders show that any combination of chalk, soap and essential oils has a marked inhibiting influence, while acid potassium tartrate has a marked stimulating influence. He found that an acid diet covering a period of eleven days—acid being taken in some form, as acid sauces on meats, acid fruits for dessert and vegetables containing some acid content—gradually increased the alkaline index from 1.73 to 4.81.

We know that very frequently chemical reactions are instantaneous. This can easily be proven by experiments with the test tube, as for instance in the reaction between an acid and a base producing a colored salt. In a similar manner reactions may take place instantly in the mouth. Acids may be present in the mouth as the result of their being taken in as food or as the result of the fermentation of sugars, the most common being the conversion of dextrose ($C_6H_{12}O_{16}$) into lactic acid $2C_3H_6O_3$. This is the reaction which probably takes place in the mouths of those who are constantly eating candy, and a similar reaction occurs in the mouths of those who eat large quantities of pastry, cakes, bread stuffs, and potatoes, wherein the starches are gradually converted into sugar and the sugar into an acid through the splitting influence of micro-organisms.

Such being the case, if it is possible to stimulate a flow of alkaline saliva for a period of 15 or 20 minutes after the ingestion of food, and if the alkalinity of the saliva can be similarly increased by regulating the diet, we have without doubt the most desirable natural method *per se* of inhibiting the progress of dental caries. In addition to this, most of the acid fruits and vegetables are fibrous in character and they have a mechanical cleansing influence upon the teeth. Again, it has been found that the mild organic acids will dissolve the plaques, thus permitting a more ready cleansing of the surfaces by the circulating saliva.

Joseph Head has found that a tooth is much more susceptible to lactic acid in an aqueous solution than it is in a saliva solution, and also that the sodium and calcium acid phosphates in the saliva are from ten to twenty times weaker in their action on the enamel than the same concentration in an aqueous solution. He maintains that the saliva exerts a protective action against the weaker acids generally.

Without having made any extensive researches on the potassium sulfocyanid question, Pickerill maintains that it is *not* an important factor in producing a natural immunity to caries. Gies is of the same opinion, stating that the salivary glands excrete rather than secrete it into the saliva; that it is a waste product, bearing no functional relation to the teeth or oral membranes. Pickerill touches upon the use of the tooth brush and D. D. Smith's method of treatment, and on the operations of the Black Club, maintaining in several instances that the accomplishment of the prevention of dental caries cannot be secured by treating the individual teeth or the individual mouth and that such result must be obtained through normal measures. He discusses the use of antiseptics and mouth washes, proving that they have an inhibiting influence upon the flow of saliva. This statement is borne out by H. Huss, a Swedish chemist, who shows that the saliva interferes with the anitseptic properties of many preparations sold for disinfecting the oral cavity. Hirata of Japan maintains that the amylolytic power of the saliva is the same at different periods of the day, that the digestive capacity remains unaltered, that it is independent of the volume secreted, of sex, and of age and that there are no significant variations in diseased conditions. In the light of Pickerill's researches it would seem that this is still an open question.

This problem is an educational measure. He maintains that there are three opportunities for preventing this disease, which we know is preventable:

1st. The teachers in primary schools This necessitates teaching the teachers the importance of the conservation of the teeth, and the method by which this can be accomplished. Then teaching the cooking teachers in the vocational schools the importance of a proper diet, thus teaching the children prophylaxis, and discouraging the eating of cakes and candies during recess periods.

2nd. Teaching the members of the medical profession the new thought in preventive medicine. When dentistry became separated from the parent profession the medical schools gave less and less instruction to their students on the mouth and teeth, with the result that today practically no instruction is given in this important branch of medicine. It is therefore necessary that the medical men, who, generally speaking, have a great deal of influence over the children for the first twelve years, particularly in prescribing their diet, should be cognizant of the prevailing conditions.

3rd. In teaching the dentists, who usually are content to repair, and have no interest in the prevention of the ravages of this disease. In reviewing six text books on dentistry—3 English, 2 American, and 1 German—there was less than one quarter of one per cent of subject matter on preventive dentistry. The dentist should not only be taught to take care of his practice in this regard, but he should be taught to teach others, because under the present conditions he is the principal medium for the dissemination of such information. In conclusion, Pickerill advocates legislation on this subject. Under conditions prevailing in Great Britian he estimates that the annual loss is eight and one-half millions of dollars, chiefly due to loss of time. He maintains that a tax on sweets and confectionery, such as is assessed on alcohol and tobacco, would have a tendency to decrease the sales of these articles, and the result would be beneficial. He believes in practically the free distribution of fruits, and as a third and last opportunity, the establishment of a thoroughly competent public health service, that will deal with preventive dentistry as it does with preventive medicine.

